# Identification of a Novel Salivary Four-miRNA Signature for Non-Invasive Diagnosis of Oral Squamous Cell Carcinoma

**DOI:** 10.3390/ijms262311373

**Published:** 2025-11-25

**Authors:** Alessia Ciringione, Giovanni Lilloni, Lucas Moron Dalla Tor, Giuseppe Perlangeli, Federica Rizzi, Tito Poli

**Affiliations:** 1 Laboratory of Biochemistry, Molecular Biology and Oncometabolism, Department of Medicine and Surgery, University of Parma, Via Volturno 39, 43125 Parma, Italy; alessia.ciringione@unipr.it; 2 Maxillo-Facial Surgery Unit, Head and Neck Department, University Hospital of Parma, Via Gramsci 14, 43125 Parma, Italy; giovanni.lilloni@unipr.it (G.L.); gperlangeli@ao.pr.it (G.P.); tito.poli@unipr.it (T.P.); 3 Department of Medicine and Surgery, University of Parma, Via Volturno 39, 43125 Parma, Italy; lucas.morondallator@unipr.it; 4 National Institute of Biostructure and Biosystems (INBB), 00136 Rome, Italy

**Keywords:** oral squamous cell carcinoma, OSCC diagnosis, salivary microRNAs, non-invasive biomarkers, OSCC management, liquid biopsy

## Abstract

Oral squamous cell carcinoma (OSCC) is the most common type of oral cancer, often diagnosed at advanced stages due to the lack of early symptoms and limitations of current invasive diagnostic methods. Salivary microRNAs (miRNAs) have emerged as promising non-invasive biomarkers for early detection. This study evaluated the diagnostic potential of ten miRNAs, selected from literature, in saliva samples from 30 OSCC patients and 30 healthy controls. The workflow included RNA extraction, reverse transcription, qRT-PCR amplification, and data normalization using the mean expression of the two most stable miRNAs identified across and within groups. Five miRNAs showed significant differential expression: miR-21 and miR-424 were upregulated, while miR-31, miR-146a, and let-7a were downregulated in OSCC patients. Receiver operating characteristic (ROC) curve analysis indicated moderate individual diagnostic power (AUC 0.658–0.720). A multivariate logistic regression combining miR-21, miR-31, miR-146a, and miR-424 yielded an AUC of 0.959, 96.7% specificity, and 86.7% sensitivity. Although limited by sample size, this study provides the first step for larger validation studies aimed at confirming the diagnostic utility of our salivary four-miRNA signature as a cost-effective and minimally invasive diagnostic tool for OSCC.

## 1. Introduction

Oral squamous cell carcinoma (OSCC) accounts for approximately 90% of oral malignancies, arising from the neoplastic transformation of the epithelium of the oral cavity [1]. Globally, it is the 16th most frequent cancer by incidence and the 15th by mortality. In Italy, approximately 5000 new cases and 1800 deaths are reported each year [2]. It is a multifactorial disease with oral potential malignant disorders and heavy tobacco and alcohol consumption being the major risk factors [3,4]. OSCC pathogenesis is driven by the accumulation of mutations in various oncogenes and tumor suppressor genes, progressing through distinct stages, including hyperplasia, dysplasia, carcinoma in situ, and ultimately metastasis [5]. According to the National Comprehensive Cancer Network (NCCN), OSCC can develop in different anatomic subsites of the oral cavity, including buccal mucosa, alveolar ridge, tongue, hard palate, retromolar trigone, floor of the mouth, and mucosa of the lips [6]. Despite advances in prevention, diagnosis, and therapeutic strategies, OSCC remains a major public health challenge, responsible for about 4.6% of cancer-related deaths worldwide [7]. The current gold standard for diagnosis involves a clinical examination followed by a tissue biopsy and imaging assessment, which also allows for staging according to the TNM (Tumor, Node, Metastasis) classification system [8]. Although this approach is effective, it can be invasive and uncomfortable for patients. Additionally, early-stage disease is often asymptomatic or exhibits non-specific symptoms, which can result in delayed diagnosis and treatment. Therefore, traditional OSCC management would take advantage from implementation of non-invasive, rapid, and cost-effective sampling methods such as liquid biopsy. This technique allows for an early detection of tumor-derived components in different biological fluids, including blood, urine, saliva, and cerebrospinal fluid, providing a real-time picture of tumor dynamics and progression [9]. Several biomarkers can be identified by liquid biopsy, such as circulating tumor cells (CTCs), circulating tumor DNA (ctDNA), tumor-derived extracellular vesicles (EVs), and circulating free RNA (cfRNA) [10]. Compared to blood-based biofluids such as plasma or serum, saliva offers a more localized and disease-relevant source of biomarkers for OSCC, as it is in direct contact with the tumor site and captures tumor-derived molecules shed into the oral cavity. Moreover, its collection is simple, painless, and cost-effective, making it a highly accessible tool for broader population screening [11].

In this context, the term “salivaomics” has been introduced to reflect the idea that saliva could be studied in the same high-throughput way as other biological fluids or tissues in the “-omics” era of biology [12]. Recent studies have highlighted that salivary non-coding RNAs (ncRNAs), including microRNAs (miRNAs), long non-coding RNAs (lncRNAs), and circular RNAs (circRNAs), hold particular promise as non-invasive biomarkers for OSCC detection and prognosis. These molecules not only reflect intrinsic tumor biology but also capture dynamic interactions between the tumor and its microenvironment, including the oral microbiota [13,14,15].

MiRNAs are small, ncRNAs involved in a wide range of biological processes, including cell differentiation, proliferation, apoptosis, and stress response [16]. By binding to complementary sequences on target messenger RNAs (mRNAs), they either inhibit translation or promote mRNA degradation, thereby modulating protein production [17]. Many of these miRNAs can promote or repress cancer development acting as “oncomirs” or tumor-suppressive miRNAs, respectively [18]. Accumulating evidence shows that miRNAs play a key role in the development and progression of OSCC, emerging as promising diagnostic/prognostic biomarkers as well as novel therapeutic targets. Over the last 15 years many studies aimed at identifying differentially expressed miRNAs in OSCC patients’ tissue and serum, however the development of non-invasive miRNAs salivary biomarkers remains an underexplored field of research. Existing studies are limited by small sample sizes, heterogeneity in patient cohorts, and variability in saliva collection and processing methods, all of which hamper reproducibility and clinical translation. In addition, identifying suitable endogenous controls or reference miRNAs for normalization in saliva samples is challenging due to the absence of universally stable miRNAs. Addressing these gaps is essential to establish reliable salivary miRNA signatures for early OSCC detection.

In the present study, we aimed to identify a novel salivary miRNA-based diagnostic signature for OSCC. To this end, we selected through a bibliographic search a panel of 10 miRNAs (miR-16, miR-191, miR-21, miR-31, miR-136, miR-146a, let-7a, miR-27b, miR-155, and miR-424) including those reported to show altered expression in OSCC patients in at least two independent studies published over the past decade and candidate reference genes.

## 2. Results

### 2.1. Clinicopathological Characteristics of the Study Population

Table 1 summarizes the study population’s clinical and demographic data. A total of 30 patients with OSCC (n = 16 females, 53%; n = 14 males, 47%) were included, along with 30 healthy controls (n = 18 females, 60%; n = 12 males, 40%). The OSCC cohort median age was 68 with a range of 35-87, while the healthy group had a median age of 60 with a range of 28-81. Regarding social history, 19 out of 30 patients (63.3%) and 8 out of 30 healthy subjects (26.6%) were current or former tobacco smokers. According to the TNM classification, most patients were diagnosed with stage IV disease (n = 14, 47%), followed by stage III (n = 5, 17%), stage II (n = 4, 13%), and stage I (n = 4, 13%). Regarding tumor location, 14 patients (47%) had a diagnosis of mandible OSCC, followed by the tongue/pelvis (n = 9, 30%), cheek (n = 4, 13%), and maxilla (n = 3, 10%).

### 2.2. RNA Concentration and Quality Assessment

The yield and quality of the extracted RNA from whole saliva were consistently adequate to support downstream molecular analyses for all collected samples. The average values of RNA concentration (ng/μL) and purity, assessed by the A260/280 ratio, are reported in Table 2.

### 2.3. miRNA Expression Levels in OSCC Patients and Healthy Controls Groups

Seven out of ten miRNAs -miR-21, miR-31, miR-16, miR-191, miR-146a, miR-424, let-7a- were successfully amplified. We used three different methods to identify the most suitable reference genes, namely NormFinder, geNorm and RefFinder. NormFinder identified miR-191 as the most stable gene (stability value = 0.37), consistently across both groups, followed by miR-16 (stability value = 0.89). In parallel, geNorm ranked miR-191 and miR-16 as the most stable pair in the overall dataset, yielding an M-value of 1.56, a value compatible with the intrinsic variability of biomarkers in biological fluids. RefFinder confirmed miR-191 and miR-16 as the most stable endogenous controls, supporting their selection as normalizers (Figure S1). Accordingly, the geometric mean of miR-191 and miR-16 was used to normalize the raw expression data of the remaining miRNAs.

**Figure 1:**
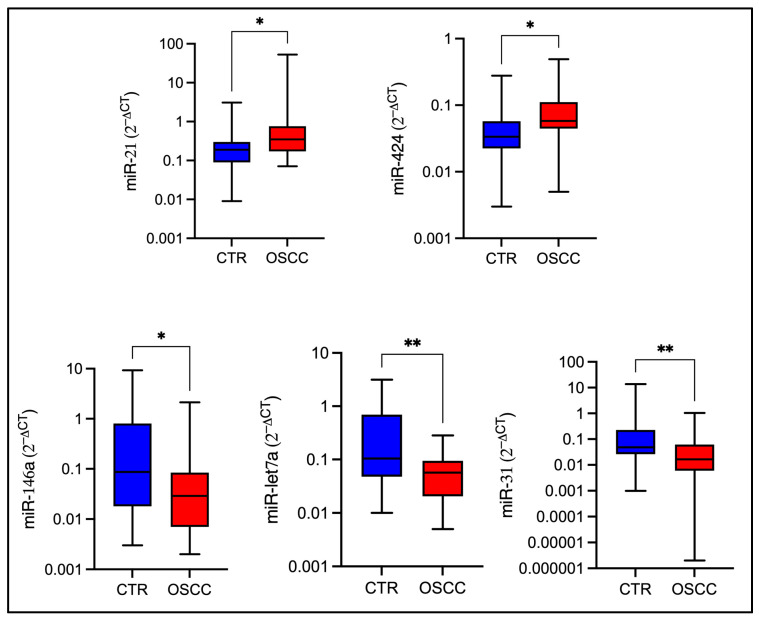
Comparison of miRNAs expression among OSCC subjects and controls. Box plots show median, interquartile ranges, and minimum-maximum values for each miRNA in OSCC patients and healthy controls. The Y axis reports the log2^−∆Ct^. Statistical significance was determined by the non-parametric Mann–Whitney U-Test (* *p*≤ 0.05; ** *p* ≤ 0.01).

OSCC patients exhibited significantly higher salivary levels of miR-21 (*p* value = 0.0116) and miR-424 (*p* value = 0.0163), and significantly lower levels of miR-31 (*p* value = 0.0029), miR-146a (*p* value = 0.0188), and let-7a (*p* value = 0.0083), compared to healthy controls (Figure 1).

### 2.4. Assessment of the Diagnostic Potential of Differentially Expressed miRNAs

**Figure 2:**
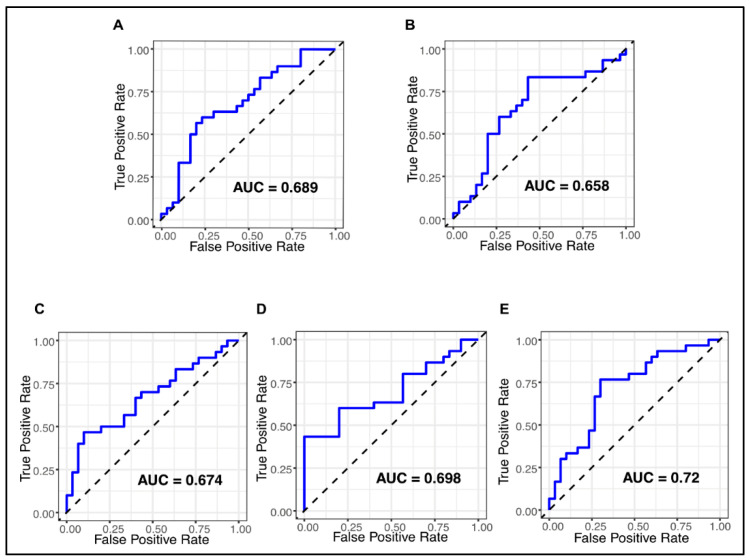
Diagnostic values of five salivary miRNAs in patients with OSCC. ROC curves of salivary miR-21 (**A**), miR-424 (**B**), miR-146a (**C**), miR-let7a (**D**), and miR-424 (**E**) to differentiate OSCC from healthy controls.

To assess the diagnostic performance of the five salivary miRNAs in distinguishing OSCC patients from healthy controls, ROC curve analyses were conducted. When evaluated individually, miR-21, miR-31, miR-146a, miR-let7a, and miR-424 demonstrated moderate potential diagnostic value, with AUCs of 0.689, 0.720, 0.674, 0.698, and 0.658, respectively (Figure 2).

**Figure 3:**
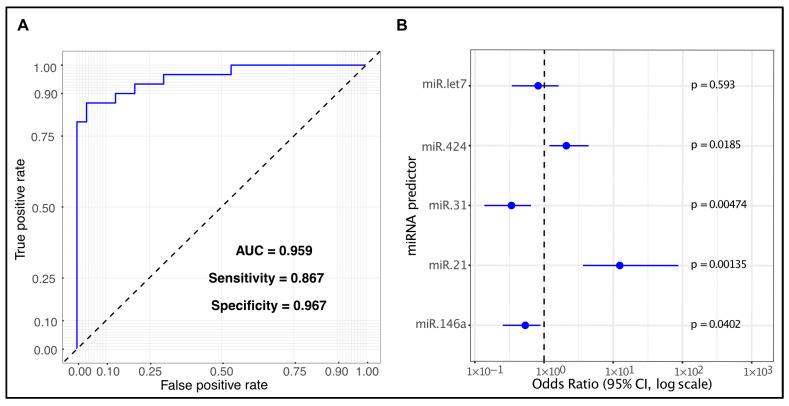
Diagnostic performance of the five salivary miRNA panel. ROC curve of the five-miRNA panel showing an AUC of 0.959, a specificity of 96.7%, and a sensitivity of 86.7% (**A**). Forest plot of the multivariate model displaying odds ratio (OR) with 95% confidence intervals for each miRNA. Dots indicate the estimated OR, bars the confidence intervals, and the dashed vertical line marks the null value (OR = 1) (**B**).

To examine their combined diagnostic power, the expression levels of all five miRNAs were integrated into a multivariate logistic regression model, yielding an AUC of 0.959 (95% CI [DeLong] = 0.9135–1; 95% CI [bootstrap] = 0.9044–0.9945), a specificity of 96.7%, and a sensitivity of 86.7% (Figure 3). All the multivariate logistic regression data are available in Supplementary Table S1.

**Figure 4:**
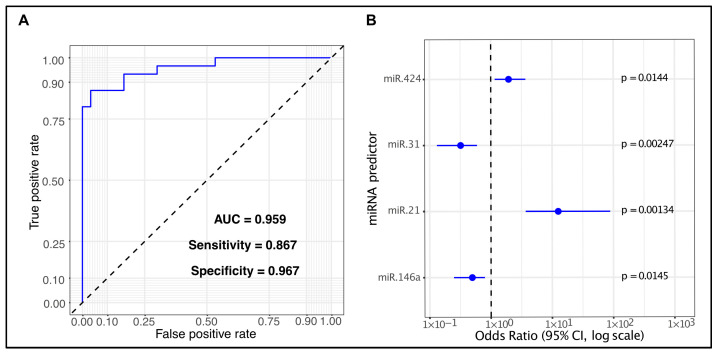
Diagnostic performance of the four salivary miRNA panel. ROC curve of the four-miRNA panel showing the identical performance metrics of the full model (**A**). Forest plot of the multivariate model displaying odds ratio (OR) with 95% confidence intervals for each miRNA. Dots indicate the estimated OR, bars the confidence intervals, and the dashed vertical line marks the null value (OR = 1) (**B**).

Since only four of the five miRNAs were statistically significant in this model, we tested whether the non-significant miRNA (miR-let7a) could be removed without negatively affecting the AUC. The four-miRNA regression model, obtained excluding miR-let7a, produced identical performance metrics, AUC = 0.959 (95% CI [DeLong] = 0.9135–1; 95% CI [bootstrap] = 0.9044–0.9956), specificity of 96.7%, and a sensitivity of 86.7% (Figure 4). The linear predictor derived from the four-miRNA logistic regression model for discriminating OSCC cases from healthy controls was expressed as follows: logit (*p* = OSCC/healthy) = −0.918 + 2.529 * miR-21 − 1.137 * miR-31 − 0.704 * miR-146a + 0.67 * miR-424. All the multivariate logistic regression data are available in Supplementary Table S2.

### 2.5. Pathway Analysis of the Four Salivary miRNA Signature

**Figure 5:**
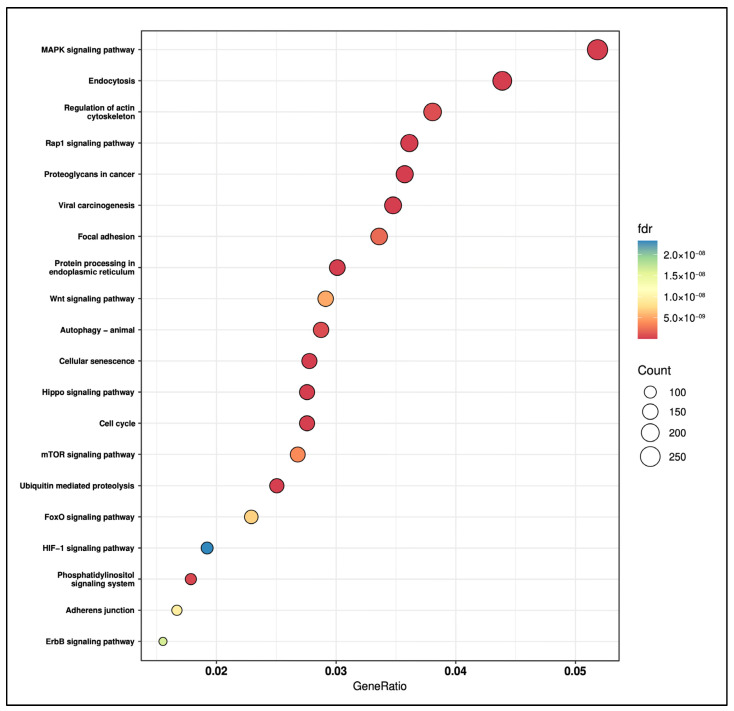
The top 20 significantly enriched KEGG pathways identified through ORA of experimentally validated target genes. The color scale represents the FDR, and the bubble size indicates the number of genes enriched in each pathway.

To explore the biological significance of the identified salivary miRNA signature, we performed an over-representation analysis (ORA) on the experimentally validated target genes for the four selected miRNAs. A total of 97 KEGG pathways were significantly enriched (false discovery rate, FDR < 0.05; see Table S3). The enriched landscape encompassed multiple signaling axes and cellular programs widely implicated in oral carcinogenesis, including the MAPK signaling cascade, the Wnt/β-catenin pathway, the PI3K/Akt/mTOR axis, as well as pathways involved in cell adhesion and cytoskeletal dynamics, such as focal adhesion and actin-cytoskeleton regulation. Figure 5 shows the top 20 significantly enriched KEGG pathways.

## 3. Discussion

The early detection of malignancies remains a critical component of cancer screening, particularly in oral squamous cell carcinoma (OSCC). However, current diagnostic gold standards are frequently invasive and may lack sensitivity in the early stages of disease progression, when OSCC typically remains clinically silent, or in patients with widespread oral cavity dysplasia. These limitations underscore the need for novel, reliable, and minimally invasive biomarkers capable not only of enabling timely diagnosis, but also of supporting patient follow-up, thereby facilitating the early detection of relapse at pre-symptomatic stages. Circulating microRNAs (miRNAs) detectable in body fluids such as plasma, serum, and saliva have emerged as highly promising non-invasive biomarkers. Given their regulatory roles in gene expression and cell signaling, miRNAs hold significant potential not only for the early detection of OSCC but also for prognostic assessment, therapeutic targeting, and molecular classification of the disease.

In this study, based on literature evidence from the last decade, we selected a panel of ten OSCC-related miRNAs (see Section 4 for selection criteria). Among these, seven were successfully amplified in our cohorts. NormFinder and GeNorm were used to identify the most stable reference genes across our groups. Accordingly, we selected miR-16 and miR-191 as endogenous controls. The expression levels of the remaining candidate diagnostic miRNAs were then normalized using the geometric mean of these two endogenous controls and calculated as 2^−ΔCt^, allowing direct comparison between OSCC patients and healthy controls.

We observed that the expression of miR-21 and miR-424 was significantly higher in the OSCC group compared with the control group. This finding is in line with recent miRNA expression profiling studies performed on tongue and buccal mucosa samples, which reported similar differences relative to corresponding normal tissues [19]. Both miRNAs showed moderate but reliable discriminatory power between OSCC and non-cancer controls (AUC of 0.689 for miR-21 and 0.658 for miR-424). MiR-21 is among the most extensively studied oncomiRs, as it is consistently overexpressed in many cancer types compared with normal tissue [20]. It is mainly linked to cell proliferation and metastasis, through the regulation of several tumor suppressor genes, such as programmed cell death protein 4 (PDCD4), phosphatase and tensin homolog (PTEN), metalloproteinase inhibitor 3 (TIMP3), tropomyosin 1 (TPM1), serpin peptidase inhibitor/clade B (SERPINB5) coding for Maspin [21]. Increased expression of miR-21 in OSCC tissue samples has also been related to advanced disease stage and lower survival [22,23]. Moreover, it has been found significantly overexpressed in CD44+ cells compared to CD44− cells, suggesting a role in stemness maintenance and regulation of cancer stem cell properties in oral cancer [23]. MiR-424 has been previously described as both an oncogenic and tumor suppressor gene depending on the tissue context [24]. Specifically, in OSCC tissues and serum samples, it was found to be upregulated compared with non-cancerous cases [25,26]. Li et al. showed that miR-424 promotes epithelial-to-mesenchymal transition and migration in tongue squamous cell carcinoma cells by targeting the transforming growth factor type III receptor (TGFBR3) [27]. However, additional studies are required to clarify the precise role played by miR-424 in oral cancer.

We also observed a significant downregulation of miR-31, miR-146a, and miR-let-7a in OSCC salivary samples. These miRNAs showed moderate but consistent diagnostic potential (AUC of 0.72 for miR-31, 0.674 for miR-146a, and 0.698 for let-7a). MiR-31 modulates several biological pathways, exerting multifaceted roles in human tumors. Its regulatory activity is both spatially and temporally specific, allowing it to modulate distinct target genes depending on the cellular context. Examples include BAP1 in cervical and lung cancer [28,29], CDK1 in bladder cancer [30], E2F2 in ovarian cancer [31], and RHOA, VEGF, NUMB, among others, in oral cancer [32]. A few studies reported elevated miR-31 levels in tumor tissue and in body fluids (saliva, serum/plasma) of OSCC patients although the association between this miRNA and clinical characteristics remains unclear [33,34,35]. In contrast, our findings indicate a significant downregulation of miR-31 in salivary samples from OSCC patients. These discrepancies may reflect differences in saliva processing, as we analyzed whole saliva rather than extracellular vesicles, pellets or supernatant fractions. Furthermore, it is noteworthy that the liquid biopsy was conducted at the time of diagnosis, and all patients presented non-metastatic OSCC and were treatment-naive. In line with our data, a tumor-suppressive role of miR-31 has been reported by Jung et al., who suggested that miR-31 regulates the cell cycle and proliferation of OSCC cells by inhibiting cyclin D1 and c-MYC expression [36]. Moreover, in line with our observation, downregulation of miR-31 has been observed in several cancer types, including glioma, leukemia, melanoma, and mesothelioma [37].

MiR-146a is primarily involved in the regulation of inflammatory and innate immune response by targeting TNF receptor-associated factor 6 (TRAF6) and interleukin IL-1 receptor-associated kinase 1 (IRAK1) genes [38]. It has also been described as a pleiotropic regulator of carcinogenesis acting either as a tumor suppressor or as an oncogene depending on the cellular context [39]. Evidence regarding its role in oral cancer remains limited and somewhat contradictory. While some studies have reported miR-146a overexpression in OSCC tissues and cell lines, where it enhanced cell migration and invasion [40,41], other investigations have shown reduced expression levels. For instance, in agreement with our findings, Lerner et al. demonstrated low expression of miR-146a in both blood and tissue samples from oral cancer patients, supporting its role as a tumor suppressor miRNA [42]. We believe that the inconsistency in miR-146a expression patterns across different studies may primarily arise from the inherent challenges in comparing data generated under heterogeneous experimental conditions. Specifically, differences in data normalization and quantification strategies, variability in cohort size and composition, as well as heterogeneity in patients’ clinical histories and enrollment criteria, are likely to exert a profound influence on the outcomes. In addition to methodological aspects, biological factors may also contribute to this divergence. In saliva, miRNA stability can be influenced by their localization in extracellular vesicles, which offer protection from enzymatic degradation. Differences in the relative abundance of extracellular vesicles in the analyzed sample, could therefore affect the detectable expression levels of specific miRNAs, including miR-31 and miR-146a [13,43]. MiR-let-7a has been described as a tumor suppressor in several human cancers by targeting genes involved in cell proliferation [44,45]. Consistent with our findings, multiple studies have reported lower miR-let-7a expression in OSCC tissue and salivary samples compared with precancerous lesions and healthy controls [46,47]. Recently, Luo et al. demonstrated that its downregulation promoted cellular proliferation, invasion, and migration via the miR-let-7a/c-Myc/MAPK/ERK signaling pathway in different OSCC cell lines [48]. Nevertheless, the complete spectrum of miR-let-7a-specific targets in OSCC has yet to be fully elucidated.

To evaluate the combined diagnostic value of all selected miRNAs, we integrated their expression levels into a single multivariate logistic regression model. We observed an AUC of 0.959, with a specificity of 96.7% and a sensitivity of 86.7%. Interestingly, miR-let-7a did not reach statistical significance, suggesting its poor association with OSCC. After its removal, the reduced model based on four miRNAs showed identical performance metrics compared to the full model.

Beyond their diagnostic performance, we performed an over-representation analysis (ORA) on experimentally validated targets of the miRNA signature to identify enriched signaling pathways implicated in oral tumor initiation and progression. Notably, the MAPK, Wnt/β-catenin, and PI3K/Akt/mTOR cascades, all central regulators of cell survival, proliferation, and migration in OSCC [49], were among the most significantly overrepresented pathways. In addition, we found pathways involved in focal adhesion and actin-cytoskeleton regulation, that play a well-established role in promoting invasion and metastatic dissemination in OSCC [50]. The enrichment of the HIF-1 signaling pathway is also biologically consistent, as HIF-1α acts as a key regulator of metabolic adaptation in OSCC, promoting glycolysis and nutrient-utilization programs under hypoxia [51]. Furthermore, the identification of Rap1, Hippo, and FoxO signaling pathways, widely implicated in tumorigenesis across multiple cancer types, indicates additional regulatory axes whose specific contributions to OSCC remain underexplored.

Finally, to facilitate comparison with previously published signatures, Table 3 summarizes studies from the past decade that have reported combinations of differentially expressed miRNAs capable of distinguishing OSCC patients from healthy controls. This comparative overview offers a broader perspective on the evolving role of circulating miRNA panels in OSCC diagnosis. Although only a limited number of studies have focused specifically on saliva, the emerging picture consistently highlights the potential of this biofluid as a minimally invasive diagnostic source. Across published investigations, diagnostic performance has varied considerably, largely due to differences in cohort composition, the statistical approaches used to generate the panels, and the number of miRNAs included in the final signature. In this landscape, the study by Romani et al. [52] represents a structured and coherent approach that integrates genome-wide profiling with independent validation and prognostic assessment, constituting a well-established methodological framework in the context of salivary biomarker development. Building on these considerations, the performance achieved by our four-miRNA panel places it among the most effective saliva-based diagnostic signatures reported so far. Its AUC of 0.959 aligns closely with the highest-performing models and exceeds the accuracy obtained in several other salivary studies. The small size of our miRNA panel, together with the strong biological coherence revealed by pathway enrichment analysis, further supports its translational potential. Future investigations may also explore potential complementarity between our signature and the miRNAs identified by Romani et al., to determine whether combined models offer incremental diagnostic benefit.

## 4. Materials and Methods

### 4.1. Patients’ Cohort and Sample Collection

The present study was designed as a mono-institutional, prospective, non-randomized study involving the enrollment of patients with primary, non-metastatic OSCC treated at the Maxillofacial Surgery Unit of the University Hospital of Parma, as well as the recruitment of healthy volunteers. The study was approved by the local ethics committee and conducted in accordance with the principles of the Declaration of Helsinki.

Inclusion criteria were as follows: adult patients diagnosed with non-metastatic OSCC (TNM stages I, II, III, IVa, and IVb), histologically confirmed by biopsy, eligible for curative treatment, and willing to participate in the study by providing written informed consent.

Exclusion criteria included a previous history of head and neck cancer or other malignancies (except for surgically treated cervical carcinoma in situ, breast carcinoma in situ, prostate carcinoma stages T1a-T1b, and cutaneous basal cell carcinoma), non-squamous histology, and metastatic disease (TNM stage IVc).

To determine the minimum cohort size required to reliably assess the diagnostic performance of candidate miRNA biomarkers, we conducted an a-priori power analysis using the power.roc.test function from the pROC package in R (version 1.19.0.1). The analysis was performed assuming a significance level of α = 0.05, a case–control ratio of 1:1, an expected area under the curve (AUC) of 0.70, and a null AUC (AUC_0_) of 0.50, representing no discriminative ability. With a target statistical power of 80% to detect this effect size, the estimated required sample size was 60 subjects, comprising 30 cases and 30 controls.

Accordingly, 60 subjects have been enrolled, including 30 patients with OSCC and 30 healthy volunteers. All 60 individuals underwent liquid biopsy from saliva samples at the time of diagnosis. To avoid contamination, they were instructed not to drink, eat, or smoke in the two hours prior to sample collection, and to brush their teeth beforehand. Saliva was collected through passive drooling in 50 mL sterile tube and immediately transferred to the Laboratory of Biochemistry, Molecular Biology and Oncometabolism, where they were stored at −80 °C for a maximum of 3 weeks until RNA extraction.

### 4.2. Selection of Candidate miRNAs as Salivary Biomarkers for OSCC

We performed a PubMed literature search limited to the last 10 years to identify miRNAs with altered expression in OSCC patients in at least two independent studies, by using the keywords “OSCC” and “miRNA”. We selected a panel of 10 miRNAs, including candidate reference genes (Table 4).

### 4.3. RNA Extraction

Frozen whole salivary samples were thawed at room temperature, divided into 400 μL aliquots, and directly processed for RNA extraction. The RNA Clean & Concentrator™ -5 kit (Zymo Research, Irvine, CA, USA) was employed according to the manufacturer’s instructions. Briefly, 1 mL of TRIzol LS Reagent^®^ optimized for liquid samples (Thermo Fisher Scientific, Waltham, MA, USA) was added to each sample, incubated at room temperature for 10 min, and mixed with 200 μL of chloroform (PanReac AppliChem, ITW Reagents, Darmstadt, Germany). Samples were then centrifuged at 14,000 rpm for 30 min at 4 °C. After moving the upper aqueous layers into new RNase-free tubes, an equal volume of ethanol absolute (VWR^®^, Fontenay-sous-Bois, France) was added. Then, samples were transferred into Zymo-Spin™ IC Columns kit for RNA clean-up and concentration and centrifuged at 13,000 rpm for 30 s. After discarding the flow-through, 400 μL of RNA Prep Buffer were added to the columns, centrifuged at 13,000 rpm for 30 s. Following two washes with RNA Wash Buffer, columns were transferred into RNase-free tubes, and the RNA was eluted into 15 μL of DNase/RNase-Free Water and stored at −80 °C until use. RNA concentration was measured using the NanoPhotometer^®^ N50 (Implen GmbH, München, Germany). The purity of the isolated RNA was monitored by the ratio of optical absorption values at the wavelengths of 260 and 280 nm.

### 4.4. cDNA Synthesis

Reverse transcription was performed using the TaqMan Advanced miRNA cDNA Synthesis Kit (Thermo Fisher Scientific, Waltham, MA, USA) according to the manufacturer’s instructions. Briefly, 10 ng of total RNA per sample was mixed with 3 μL of Poly(A) Reaction Mix, then incubated at 37 °C for 45 min and 65 °C for 10 min. Next, 10 μL of the Ligation Reaction Mix was transferred to each sample and incubated at 16 °C for 1 h. Finally, 15 μL of the Reverse Transcription Reaction Mix was mixed with each sample and incubated at 42 °C for 15 min and 85 °C for 5min. The reaction products were stored at -20 °C for maximum 2 months until quantitative Real-Time PCR (qRT-PCR) analysis.

### 4.5. Quantitative Real-Time PCR

Before performing the qRT-PCR, 5 μL of the reverse transcription products were mixed with 45 μL of the miR-Amp Reaction Mix. This pre-amplification reaction included an initial step at 95 °C for 5 min, followed by 14 cycles of 3 s at 95 °C and 30 s at 60 °C, and a final step of 10 min at 99 °C. Next, each cDNA template was diluted 1:10 and mixed with the PCR Reaction Mix. The amplification was carried out on a QuantStudio3 platform (Thermo Fisher Scientific, Waltham, MA, USA) and included an initial step at 95 °C for 30 s, followed by 40 cycles of 1 s at 95 °C and 20 s at 60 °C. Amplification reactions were performed at least in duplicate on 96-well plates, and at least one negative control was included for each run. CT values differing by more than 0.5 between duplicate samples were discarded, and amplifications were repeated. The relative expression of target miRNA in each sample was calculated using the 2^−∆Ct^ method following normalization with selected reference miRNAs.

### 4.6. qRT-PCR Data Normalization

To identify the most reliable reference genes, we evaluated the expression stability of all candidate miRNAs using the NormqPCR package in R (version 1.56.0) [73], which implements both the geNorm and NormFinder algorithms. NormFinder [74] estimates both intra- and inter-group variability, while geNorm [75] ranks candidate genes based on the average pairwise variation in their expression profiles. Raw Ct values were converted into relative quantities (2^−Ct^), and patient/control grouping was included in the NormFinder analysis. To independently validate these results, the same set of candidate reference miRNAs was additionally analyzed using the RefFinder online platform [76], which integrates the major current computational tools used for reference gene evaluation: geNorm, NormFinder, BestKeeper, and the comparative ΔCt method. Based on the rankings produced by each individual algorithm, the platform assigns a weight to every gene and computes the geometric mean of these weights to generate a final consensus stability ranking.

### 4.7. Statistical Analysis

The data were analyzed using GraphPad Prism 9 and R (version 4.4.2) software. Groups’ comparisons were performed using the Mann-Whitney U test and a *p*-value ≤ 0.05 was considered significant. All available clinical variables were evaluated using principal component analysis (PCA) considering up to five principal components, and no evidence of batch effects was observed. The diagnostic performance of single miRNAs was assessed through receiver operating characteristic (ROC) curve analysis. To evaluate the combined predictive value of multiple miRNAs, we integrated the expression levels of all selected miRNAs into a single multivariate logistic regression model. Each miRNA’s expression (measured as −ΔCt values) was used as a continuous predictor, and the binary outcome (e.g., tumor vs. normal) was the response variable. The diagnostic performance of the multivariate logistic regression model was evaluated by the area under the receiver operating characteristic curve (AUC). Ninety-five percent confidence intervals (95% CI) for the AUC were obtained using both DeLong’s analytic method and a bootstrap procedure (4000 stratified resamples). The bootstrap was applied solely to estimate the uncertainty of the AUC and was not used for model validation or resampling-based performance correction. Sensitivity and specificity were computed using a threshold determined by the Youden J statistic.

### 4.8. Pathways Analysis

Experimentally validated target genes for the four selected miRNAs were retrieved using the multiMiR R package (version 1.28.0) [77]. The individual target lists were merged, and duplicate gene symbols were removed to obtain a unified set of miRNA targets. Over-representation analysis (ORA) [78] was then performed to identify pathways significantly enriched among the predicted targets. Gene-pathway annotations for the Kyoto Encyclopedia of Genes and Genomes (KEGG) [79] were obtained through the KEGGREST R package [80] and subsequently filtered according to the BRITE hierarchy (Biological Relationship and Information Transmission) [81] to include: “Metabolism, Genetic Information Processing, Environmental Information Processing, and Cellular Processes”. From the Human Diseases category, we retained pathways within the “Cancers: Overview” subcategory, excluding infectious diseases, and other non-cancer-related disease pathways. Enrichment significance was assessed using a hypergeometric test with multiple testing correction (Benjamini–Hochberg/FDR), and pathways with adjusted *p*-values < 0.05 were considered significantly enriched.

## 5. Conclusions

This study identifies a salivary four-miRNA signature (miR-21, miR-424, miR-146a, and miR-31) as a promising non-invasive biomarker for distinguishing OSCC patients from healthy individuals.

Notably, the signature achieved an outstanding diagnostic performance (AUC = 0.959, sensitivity = 86.7%, specificity = 96.7%), exceeding the effect size (AUC = 0.70) assumed in the a priori power analysis used to determine the study sample size (see Section 4). Nonetheless, we acknowledge that the relatively limited number of enrolled participants may affect the robustness and external validity of the statistical findings. This limitation will be specifically addressed in a dedicated multi-center validation study designed to evaluate the diagnostic power, reproducibility, and scalability of the miRNA assay in real-world clinical settings by including larger, independent cohorts.

Overall, this study represents the first step of a broader project aimed at refining patient risk stratification through the biological characterization of tumor aggressiveness. We believe that only a multidisciplinary approach combining histopathological analysis, imaging techniques and molecular profiling will contribute to more informed clinical decision-making in OSCC by refining early diagnostic accuracy and supporting risk-adapted therapeutic planning.

## Figures and Tables

**Table 1:** Clinicopathological characteristics of the study population.

Characteristics		OSCC Patients (n = 30)	Healthy Controls (n = 30)
Median age		68	60
Gender	F	16 (53%)	18 (60%)
	M	14 (47%)	12 (40%)
Smoke	Yes	5 (17%)	6 (20%)
	No	11 (37%)	19 (63%)
	Former *	14 (47%)	2 (7%)
OSCC stage	I	4 (13%)	NA
	II	4 (13%)	
	III	5 (17%)	
	IV	14 (47%)	
Tumor location	Tongue and pelvis	9 (30%)	NA
	Mandible	14 (47%)	
	Maxilla	3 (10%)	
	Cheek	4 (13%)	

(*) Former smokers are defined as individuals who have abstained from smoking for a minimum of six months.

**Table 2:** The mean values of RNA concentration and A260/280 ratio of the isolated RNA.

Groups	RNA Mean Concentration (ng/μL)	A260/280 Ratio
OSCC	216.02	1.88
Healthy controls	213.11	2.07

**Table 3:** Summary of diagnostic multi-miRNA panels distinguishing oral cancer from healthy subjects (2015–2025). The table reports the study cohorts, the type of biological specimen, the diagnostic performance (AUC, sensitivity, and specificity, when available) of each miRNA panel, the statistical approach used for panel construction, and the corresponding reference. Studies are listed in chronological order, from the earliest to the most recent publication.

Panel	Cohorts	Sample Type	Diagnostic Performance	Method for Panel Generation	Reference
miR-9-5p, miR-127-5p, miR-134-3p, miR-191-5p, miR-222-3p, miR-455-3p	47 HPV-negative HNSCC patients 113 healthy controls	Unstimulated whole saliva	AUC 0.82 Sensitivity: 60% Specificity: 94%	Logistic regression model	[53]
miR-9-5p, miR-134-3p, miR-210-5p, miR-455-3p, miR-196b-3p	54 HPV-positive HNSCC patients 113 healthy controls	Unstimulated whole saliva	AUC 0.80 Sensitivity: 65% Specificity: 95%	Logistic regression model	[53]
miR-150-5p, miR-423-5p	* 82 OSCC patients 50 healthy controls	Plasma	AUC 0.749 (95% CI: 0.678–0.819) Sensitivity: 70.9% Specificity: 72.8%	Logistic regression model	[54]
miR-204-5p, miR-193b-5p, miR-370-3p, miR-144-5p	195 OSCC samples 103 normal oral mucosa samples	FFPE	AUC: 0.92 (95% CI, 0.90–0.97)	Logistic regression model	[55]
miR-30a-5p, miR-769-5p	55 OSCC patients 18 healthy controls	Plasma	AUC: 1	Logistic regression model	[55]
miR-21-5p, let-7c-5p, miR-100-5p	30 OSCC patients 30 healthy controls	Oral swirl	AUC 0.86 (95% CI: 0.79–1.00)	Dysregulation score/Classification tree	[56]
miR-99a-5p, miR-31-5p, miR-138-5p, miR-21-5p, miR-375-3p	82 oral cancer patients 53 healthy controls	Serum	AUC 0.776 (95% CI: 0.695–0.857) Sensitivity: 76.8% Specificity: 73.6%	Logistic regression model	[57]
miR-24-3p, miR-21-5p, let-7c-5p, miR-99a-5p, miR-100-5p	53 OSCC patients 54 healthy controls	Oral swirl	AUC 0.8676 Sensitivity: 86.8% Specificity: 81.5%	Dysregulation score/Classification tree	[58]
miR-106b-5p, miR-423-5p, miR-193b-3p	* 28 OSCC patients 14 healthy controls	Unstimulated whole saliva	AUC: 0.923 [95% CI: 0.908–0.938] Sensitivity: 85.4% Specificity: 85.1%	Logistic regression model	[52]
miR-24-3p, miR-20a-5p, miR-122-5p, miR-150-3p, miR-4419a, miR-5100	* 40 OSCC patients 40 healthy controls	Serum	AUC: 0.844 Sensitivity: 55% Specificity: 92.5%	Fisher’s linear discriminant analysis	[59]
miR-345-3p, miR-424-3p, miR-31-5p	43 OSCC patients 44 healthy controls	Unstimulated whole saliva	AUC 0.8647 (95% CI: 0.7855–0.9439) Sensitivity: 76.7% Specificity: 85.7%	Logistic regression model	[60]
miR-31-3p, miR-139-5p, miR-30a-5p	35 OSCC lesions Matched non-cancerous tissue	Fresh tissue	AUC 0.780 (95% CI: 0.673–0.886) Sensitivity: 94.3% Specificity: 51.4%	Logistic regression model	[61]
miR-92a-3p, miR-92b-3p, miR-320c, miR-629-5p	* 23 OSCC patients 15 healthy controls	Serum	AUC 0.899 (95% CI: 0.8431–0.9547) Sensitivity: 73.9% Specificity: 97.8%	Logistic regression model	[62]
miR-125b-5p, miR-342-3p	* 65 early-stage OSCC or carcinoma in situ lesions 69 healthy controls	Serum	AUC: 0.801 Sensitivity: 74% Specificity: 74%	LASSO regression	[63]
miR-7-5p, miR-10b-5p, miR-182-5p, miR-431-5p, miR-3614-5p, miR-4707-3p, miR-215-5p, miR-486-3p	* 50 oral cancer patients 60 healthy controls	Unstimulated whole saliva	AUC: 0.954 Sensitivity: 86% Specificity: 90%	LASSO regression	[64]

* Validation phase of the study.

**Table 4:** Selected miRNAs as candidate biomarkers for OSCC along with their sequences.

miRNA	Target Sequences	References
hsa-miR-21-5p	5′-UAGCUUAUCAGACUGAUGUUGA-3′	[65]
hsa-miR-31-5p	5′-AGGCAAGAUGCUGGCAUAGCU-3′	[32]
hsa-miR-16-5p	5′-UAGCAGCACGUAAAUAUUGGCG -3′	[66]
hsa-miR-191-5p	5′-CAACGGAAUCCCAAAAGCAGCUG-3′	[66]
hsa-miR-136-5p	5′-ACUCCAUUUGUUUUGAUGAUGGA-3′	[67,68]
hsa-miR-27b-5p	5′-AGAGCUUAGCUGAUUGGUGAAC-3′	[69,70]
hsa-miR-146a-5p	5′-UGAGAACUGAAUUCCAUGGGUU-3′	[41,42]
hsa-let-7a-5p	5′-UGAGGUAGUAGGUUGUAUAGUU-3′	[46,47]
has-mir-424-3p	5′-CAAAACGUGAGGCGCUGCUAU-3′	[25,26]
has-mir-155-3p	5′-CUCCUACAUAUUAGCAUUAACA-3′	[71,72]

## Data Availability

The datasets presented in this article are not readily available because part of an ongoing study. Requests to access the datasets should be directed to federica.rizzi@unipr.it (corresponding author).

## Supplementary Materials

The following supporting information can be downloaded at: https://www.mdpi.com/article/10.3390/ijms262311373/s1.

## Abbreviations

The following abbreviations are used in this manuscript:

OSCC	Oral squamous cell carcinoma
ncRNAs	Non-coding RNAs
MiRNAs	MicroRNAs
TNM	Tumor, Node, Metastasis
qRT-PCR	Quantitative real-time PCR
PCA	Principal component analysis
ROC	Receiver operating characteristic
AUC	Area under the curve
ORA	Over-representation analysis
KEGG	Kyoto Encyclopedia of Genes and Genomes
FDR	False discover rate
