# Supplementary material for: Identification of a Novel Salivary Four-miRNA Signature for Non-Invasive Diagnosis of Oral Squamous Cell Carcinoma
Source: Int J Mol Sci. 2025 Nov 25;26(23):11373. doi: 10.3390/ijms262311373 (PMC12692314; doi:10.3390/ijms262311373)
Supplement: Supplementary file 1 [file ijms-26-11373-s001.zip › Table S1.pdf]

Table S1. Results of logistic regression model of five miRNAs

Term	Estimate(β)	Standard error	z-value	p-value	Odds ratio	Lower_95_CI	Upper_95_CI
Intercept	-0,777027797	1,859597453	-0,41784731	0,676059	0,45977051	0,009437864	17,85251599
miR-21	2,515360623	0,784692358	3,205537302	0,001348	12,3710693	3,642624451	86,92942427
miR-424	0,733430944	0,311374725	2,355460755	0,0185	2,08221232	1,187467045	4,365958958
miR-146a	-0,626415836	0,305256333	-2,05209775	0,04016	0,53450412	0,25283005	0,884546021
miR-let7a	-0,207470236	0,388479726	-0,53405679	0,593302	0,81263743	0,338895812	1,606351587
miR-31	-1,08975202	0,385840401	-2,82435954	0,004738	0,33629988	0,136243832	0,642967027
