# Supplementary material for: Identification of a Novel Salivary Four-miRNA Signature for Non-Invasive Diagnosis of Oral Squamous Cell Carcinoma
Source: Int J Mol Sci. 2025 Nov 25;26(23):11373. doi: 10.3390/ijms262311373 (PMC12692314; doi:10.3390/ijms262311373)
Supplement: Supplementary file 1 [file ijms-26-11373-s001.zip › Table S2.pdf]

Table S2. Results of logistic regression model of four miRNAs

Term	Estimate(β)	Standard error	z-value	p-value	Odds ratio	Lower_95_CI	Upper_95_CI
Intercept	-0,91773	1,827149	-0,50227	0,615475	0,399425	0,008492	14,04207
miR-21	2,528648	0,788394	3,207341	0,00134	12,53654	3,69196	88,55249
miR-424	0,669692	0,273674	2,447041	0,014403	1,953635	1,161305	3,686922
miR-146a	-0,70368	0,287941	-2,44383	0,014532	0,494763	0,248039	0,79433
miR-31	-1,13703	0,375652	-3,02681	0,002471	0,320771	0,131759	0,595324
