# Supplementary figures and images for: Identification of a Novel Salivary Four-miRNA Signature for Non-Invasive Diagnosis of Oral Squamous Cell Carcinoma
Source: Int J Mol Sci. 2025 Nov 25;26(23):11373. doi: 10.3390/ijms262311373 (PMC12692314; doi:10.3390/ijms262311373)

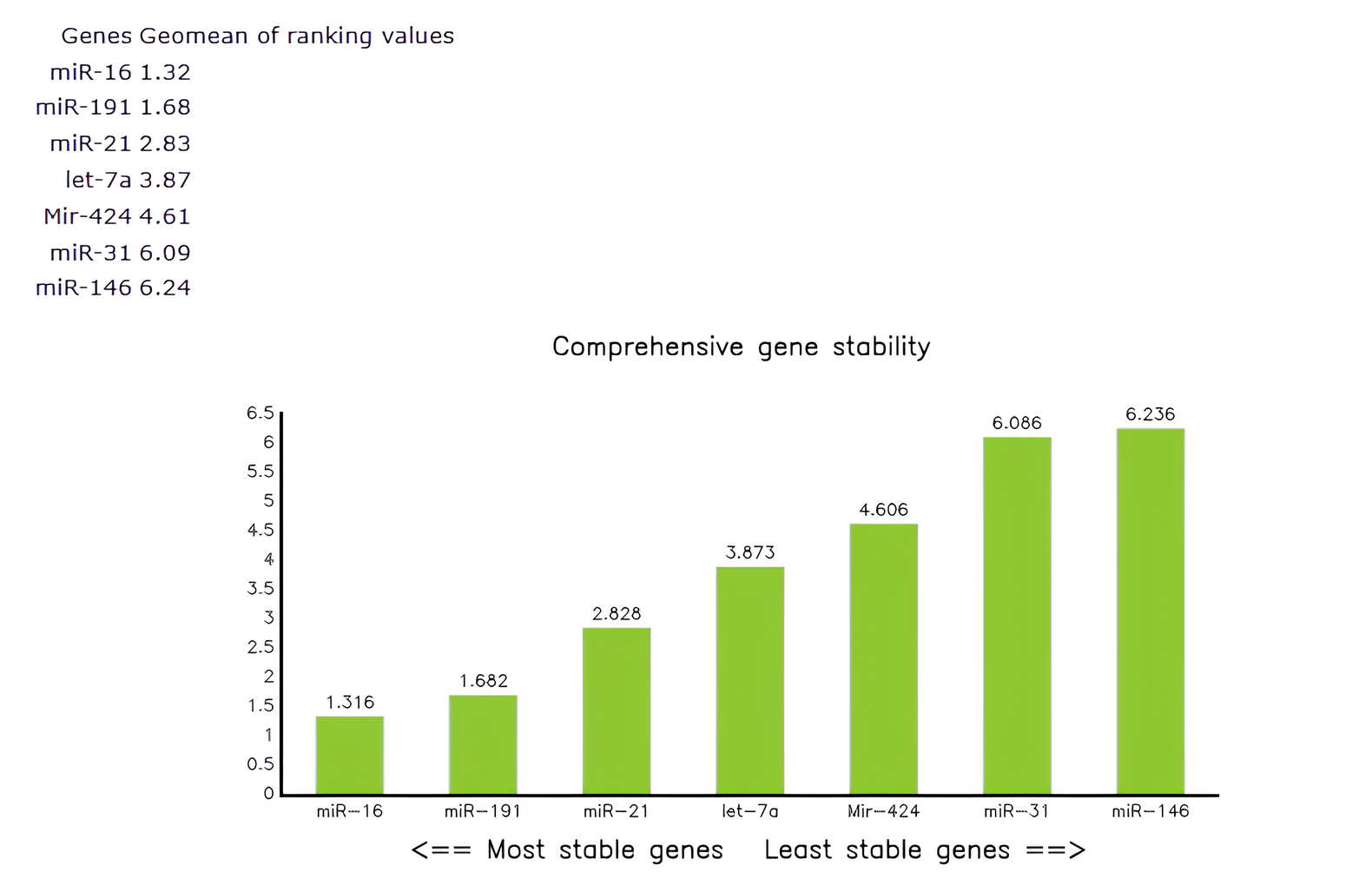

Supplement: Supplementary file 1 [file ijms-26-11373-s001.zip › Figure S1.png]
